# Retinoic Acid Inhibits Tumor-Associated Mesenchymal Stromal Cell Transformation in Melanoma

**DOI:** 10.3389/fcell.2021.658757

**Published:** 2021-04-06

**Authors:** Qi Lou, Minyi Zhao, Quanhui Xu, Siyu Xie, Yingying Liang, Jian Chen, Lisha Yuan, Lingling Wang, Linjia Jiang, Lisha Mou, Dongjun Lin, Meng Zhao

**Affiliations:** ^1^Department of Hematology, The Seventh Affiliated Hospital, Sun Yat-sen University, Shenzhen, China; ^2^Shenzhen Lansi Institute of Artificial Intelligence in Medicine, Shenzhen, China; ^3^Health Science Center, The First Affiliated Hospital of Shenzhen University, Shenzhen Second People’s Hospital, Shenzhen, China; ^4^Sun Yat-sen Memorial Hospital, RNA Biomedical Institute, Sun Yat-sen University, Guangzhou, China; ^5^Key Laboratory of Stem Cells and Tissue Engineering, Zhongshan School of Medicine, Ministry of Education, Sun Yat-sen University, Guangzhou, China; ^6^The Fifth Affiliated Hospital of Sun Yat-sen University, Zhuhai, China

**Keywords:** MSC, tumor associated MSC, retinoic acid, interleukin-17, interferon-γ, tumor microenvironment

## Abstract

Bone marrow mesenchymal stem/stromal cells (BMSCs) can be transformed into tumor-associated MSCs (TA-MSCs) within the tumor microenvironment to facilitate tumor progression. However, the underline mechanism and potential therapeutic strategy remain unclear. Here, we explored that interleukin 17 (IL-17) cooperating with IFNγ transforms BMSCs into TA-MSCs, which promotes tumor progression by recruiting macrophages/monocytes and myeloid-derived suppressor cells (MDSCs) in murine melanoma. IL-17 and IFNγ transformed TA-MSCs have high expression levels of myelocyte-recruiting chemokines (CCL2, CCL5, CCL7, and CCL20) mediated by activated NF-κB signaling pathway. Furthermore, retinoic acid inhibits NF-κB signaling, decreases chemokine expression, and suppresses the tumor-promoting function of transformed TA-MSCs by prohibiting the recruitment of macrophages/monocytes and MDSCs in the tumor microenvironment. Overall, our findings demonstrate that IL-17 collaborating with IFNγ to induce TA-MSC transformation, which can be targeted by RA for melanoma treatment.

## Introduction

Mesenchymal stem/stromal cells (MSCs) self-renew and differentiate into adipocytes, osteoblasts, and chondroblasts in bone marrow (BM) (Friedenstein et al., 1966, 1976, 1987), where BM MSCs (BMSCs) produce multiple growth factors, including SCF, CXCL12, Ang, and Wnt ligands, to support hematopoiesis (Kfoury and Scadden, 2015). Furthermore, MSCs reside in various tissues, such as liver, heart, adipose tissue, and lymph node to support their tissue homeostasis and regeneration (Uccelli et al., 2008). Additionally, tissue-resident MSCs can regulate immune response by producing various immunoregulatory molecules, such as TGF-β, NOS2, PEG2, and PD-L1 (Jiang and Xu, 2020).

MSCs are also involved in tumor progression (Pietras and Ostman, 2010). Tumor-associated MSCs (TA-MSCs) support tumor cell growth and angiogenesis by secreting multiple growth factors, such as BMP and VEGF (Beckermann et al., 2008; McLean et al., 2011). TA-MSCs also suppress immunosurveillance in the tumor microenvironment by inhibiting adaptive and innate immune cells. TA-MSCs suppress T cells by producing immune suppressive factors, such as NOS2, IDO, and PD-L1 (Ren et al., 2008). More importantly, TA-MSCs can recruit macrophages and myeloid-derived suppressor cells (MDSCs) into the tumor microenvironment through CC-chemokine receptor 2 (CCR2) ligands, including CC-chemokine ligand 2 (CCL2), CCL7, and CCL12 (Ren et al., 2012). The recruited macrophages and MDSCs further suppress immune surveillance and promote tumor growth within the tumor microenvironment to promote tumor growth (Qian and Pollard, 2010; Kumar et al., 2016).

TNFα, a proinflammatory cytokine highly expressed in tumor inflammatory environment, can transform BMSCs to TA-MSCs, which produce high-level CCR2 ligands to promote tumor growth by recruiting monocytes/macrophages (Ren et al., 2012). Interleukin 17 (IL-17) is an important pro-inflammatory cytokine secreted by CD4^+^ Th17 and CD8^+^ Tc17 cells and highly expressed in tumor microenvironment (Miossec et al., 2009). Deletion of IL-17 reduces MDSCs in tumor microenvironment and inhibits tumor growth (He et al., 2010; Wu et al., 2014). However, whether IL-17 participates in TA-MSC transformation to support tumor growth within tumor microenvironment remains unknown. Retinoic acid (RA), a metabolite of vitamin A (Cunningham and Duester, 2015), can induce differentiation of acute promyelocytic leukemia cells (de Thé, 2018). Studies suggest that RA could inhibit solid tumor growth and regulate the tumor microenvironment (Abu et al., 2005; Bolis et al., 2020; Sun et al., 2020). Here, we found that IL-17 incorporating with IFNγ transforms BMSCs into TA-MSCs to promote tumor growth, which is inhibited by RA treatment in melanoma.

## Results

### IL-17 and IFNγ Transform BMSCs Into TA-MSCs to Facilitate Melanoma Progress *in vivo*

**FIGURE 1:**
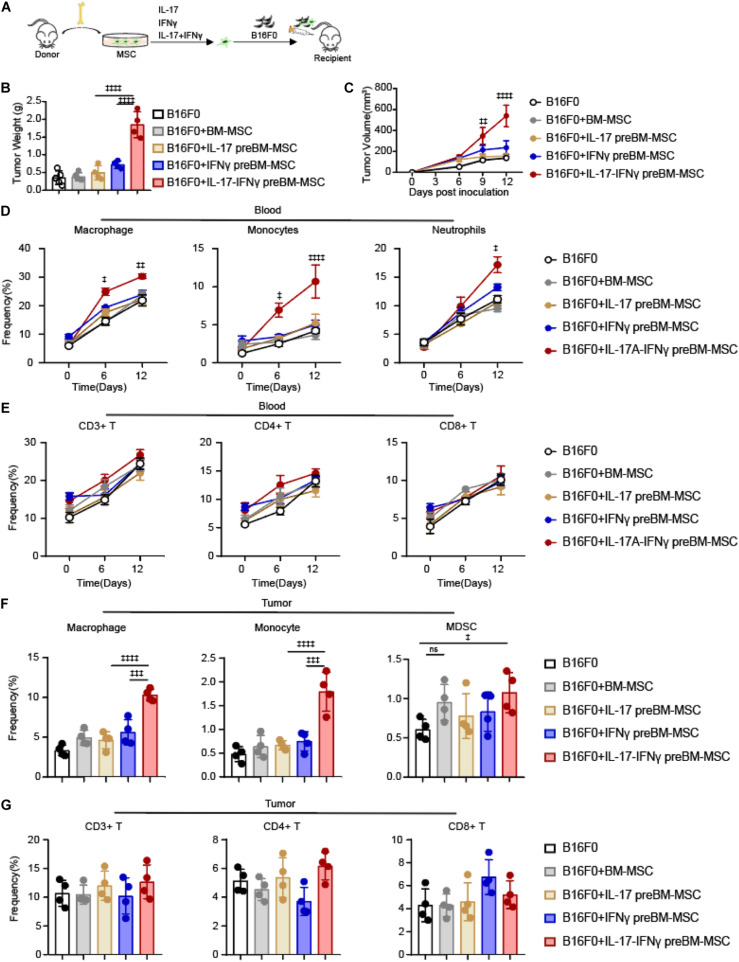
IL-17 and IFNγ transform BMSCs into TA-MSCs to facilitate melanoma progress *in vivo*. **(A)** Schematic depicting the strategy to investigate the tumor-promotion function of IL-17 and IFNγ transformed BMSCs in murine melanoma. **(B,C)** Tumor weight **(B)** and tumor growth curve **(C)** in melanoma mice inoculated with B16F0 cells alone or with IL-17 and/or IFNγ pre-treated BMSCs (*n* = 4–5). **(D,E)** The percentage of circulated macrophages, monocytes, neutrophils **(D)**, and T cells **(E)** in the peripheral blood. **(F,G)** The frequency of resident macrophages, monocytes, MDSCs **(F)**, and T cells **(G)** in the tumor tissues in melanoma mice inoculated with B16F0 cells alone or with IL-17 and/or IFNγ pre-treated BMSCs (*n* = 4). Data represent mean ± *SD* of 3 independent experiments. ^‡^*p* < 0.05, ^‡⁣‡^*p* < 0.01, ^‡⁣‡⁣‡^*p* < 0.001, ^‡⁣‡⁣‡‡^*p* < 0.0001. ns, not significant.

To explore the role of IL-17 in transforming BMSCs to TA-MSCs, we investigated the tumor growth co-engrafted with BMSCs and IL-17 transformed MSCs. We subcutaneously inoculated B16F0 melanoma cells with normal BMSCs or BMSCs pretreated with IL-17 and IFNγ, respectively, or jointly into C57BL/6 mice (Figure 1A). The B16F0 melanoma cells with BMSCs, which were pretreated with IL-17 and IFNγ jointly, gave more aggressive tumor growth compared to control B16F0 melanoma cells (5.2-fold increase in tumor weight, and 4-fold increase in tumor size). However, B16F0 melanoma cells with normal BMSCs or BMSCs treated with IL-17 and IFNγ respectively, did not show a significant difference in tumor weight or volume compared to control B16F0 melanoma cells (Figures 1B,C). This indicated that IL-17 incorporated with IFNγ to stimulate the BMSC to TA- MSC transformation, which promoted tumor growth in melanoma. Furthermore, we investigated that whether IL-17 and IFNγ transformed TA-MSCs can recruit monocytes/macrophages and MDSCs. Our FACS assay showed that the myelocytes, including macrophages, monocytes, and neutrophils, were dramatically increased in peripheral blood when melanoma mice were co-inoculated with IL-17 and IFNγ transformed TA-MSCs (1. 5-, 2. 5-, and 1.6-fold increase, respectively) (Figure 1D). However, no significant increase of circulating T cells was observed in mice co- engrafted with pretreated TA-MSCs compared to mice with control melanoma cells (Figure 1E). No significant increase of either myelocytes or T cells was observed in peripheral blood when melanoma mice were co-inoculated with normal BMSCs or BMSCs pretreated with IL-17 or IFNγ individually (Figure 1D). More importantly, the increased macrophages, monocytes, and MDSCs were observed in the tumor microenvironment (2. 5-, 3. 5-, and 1.6-fold increase, respectively) when melanoma mice were co-engrafted with IL-17 and IFNγ transformed TA-MSCs (Figure 1F). However, co-inoculation of normal BMSCs or BMSCs pretreated with IL-17 or IFNγ individually did not increase the numbers of macrophages and monocytes in the tumor microenvironment, and the BMSC induced slight increase of MDSCs was not statistically significant (Figure 1F). Furthermore, tumor-resident T cells were not regulated by control BMSCs or by transformed TA-MSCs (Figure 1G).

Overall, our observations showed that IL-17 and IFNγ jointly but not individually transformed normal BMSCs into TA-MSCs, and the IL-17 and IFNγ transformed TA-MSCs can recruit myelocytes into the tumor microenvironment to promote tumor growth.

## IL-17 and IFNγ Synergistically Increase Immunoregulatory Genes in BMSCs

**FIGURE 2:**
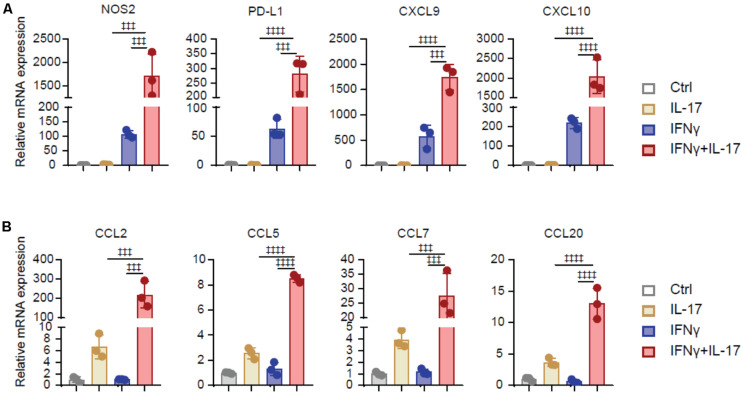
IL-17 and IFNγ synergistically increase immunoregulatory genes in BMSCs. **(A,B)** The relative mRNA expression level of immunoregulatory molecules (NOS2, PD-L1, CXCL9, and CXCL10) **(A)** and myelocyte-recruiting chemokines (CCL2, CCL5, CCL7, and CCL20) **(B)** in the BMSCs treated with IL-17 and IFNγ, respectively, or jointly. Data represent mean ± *SD* of 3 independent experiments. ^‡⁣‡⁣‡^*p* < 0.001, ^‡⁣‡⁣‡‡^*p* < 0.0001. ns, not significant.

To investigated the underlining mechanism that IL-17 and IFNγ transform BMSCs to TA-MSCs, we analyzed the expression of immunoregulatory molecules in BMSCs after IL-17 and/or IFNγ stimulation. Transcriptional analysis showed that IL-17 and IFNγ synergistically increased the expression level of immunosuppressors, such as NOS2, PD-L1, CXCL9, and CXCL10 (1, 711-, 280-, 1, 742-, and 2,035-fold increase, respectively) in transformed TA-MSCs compared to control BMSCs. IFNγ individual treatment also significantly increased the expression of immunosuppressors (106-, 62-, 573-, and 218-fold increase, respectively), however, IL-17 treatment did not show a significant effect on these immunosuppressors (Figure 2A). Furthermore, IL-17 and IFNγ synergistically stimulate a dramatic increase of myelocyte recruiting chemokines, including CCL2, CCL5, CCL7, and CCL20 (218-, 8-, 27-, and 13-fold increase, respectively) in transformed TA-MSCs, although a slight increase was observed in BMSCs after IL-17 treatment (6-, 2-, 4-, and 3-fold increase, respectively) (Figure 2B). No significant increase of myelocyte recruiting chemokines was observed in BMSCs after IFNγ treatment (Figure 2B).

Taken together, these data illustrated that IL-17 and IFNγ synergistically increased the expression of immunosuppressive factors and myelocyte recruiting factors in BMSCs, but IL17 or IFNγ individual treatment had a limited effect on TA-MSC transformation.

### RA Inhibits TA-MSC Transformation and Further Suppresses Melanoma Progress *in vivo*

**FIGURE 3:**
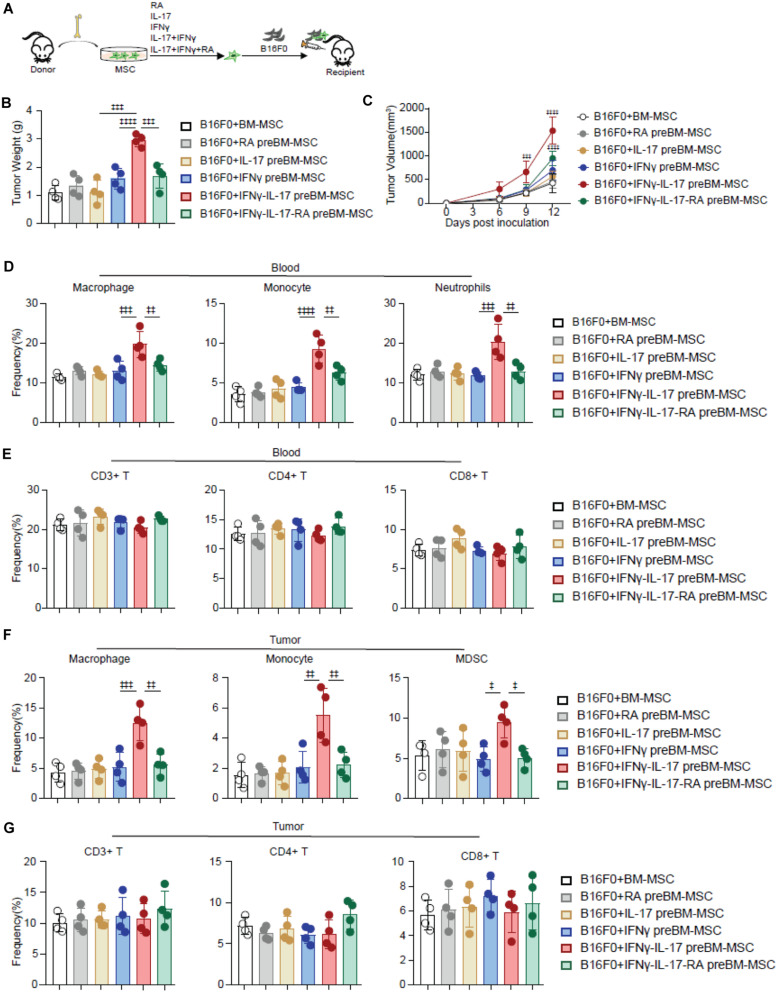
RA inhibits TA-MSC transformation and suppresses TA-MSC mediated melanoma progress *in vivo*. **(A)** Schematic depicting the strategy to investigate the inhibition role of RA on IL-17 and IFNγ mediated TA-MSC transformation. **(B,C)** Tumor weight **(B)** and tumor growth curve **(C)** of mice inoculated with B16F0 cells, and B16F0 cells with BMSCs with indicated treatment (*n* = 4–5). **(D,E)** The percentage of circulated macrophages, monocytes, neutrophils **(D)**, and T cells **(E)** in the peripheral blood. **(F,G)** The frequency of macrophages, monocytes, MDSCs **(F)** and T cells **(G)** in the tumor microenvironment of mice inoculated with B16F0 cells, and B16F0 cells with BMSCs with indicated treatment at 12 days after tumor cell inoculation (*n* = 4). Data represent mean ± *SD* of 3 independent experiments. ^‡^*p* < 0.05, ^‡⁣‡^*p* < 0.01, ^‡⁣‡⁣‡^*p* < 0.001, ^‡⁣‡⁣‡‡^*p* < 0.0001. ns, not significant.

RA inhibits Th17 differentiation (Mucida et al., 2007; Elias et al., 2008), which suggested that RA might inhibit IL-17 signaling. Therefore, we investigated whether RA regulates IL-17 mediated TA-MSC transformation. We simultaneously supplied RA during IL-17 and IFNγ mediated TA-MSC transformation, and further performed co-engrafted cell-derived xenograft experiments with B16F0 melanoma cells and transformed TA-MSCs or RA treated TA-MSCs (Figure 3A). Intriguingly, RA supplement dramatically inhibited the tumor-promoting capacity of IL-17 and IFNγ transformed TA-MSCs, which had 68% reduction in tumor weight (Figure 3B) and 53% reduction in tumor volume compared to TA-MSCs without RA treatment (Figure 3C). To explore the underline mechanism, we further analyzed the myelocytes in peripheral blood and tumor microenvironment. We surprisingly found that RA remarkably inhibited the myelocyte recruiting function of TA-MSCs, with 64% decrease of macrophages, 53% decrease of monocytes, and 89% decrease of neutrophils in peripheral blood (Figure 3D). More importantly, RA- treated TA-MSCs completely failed to recruit macrophages, monocytes, and MDSCs into the tumor microenvironment (85, 83, and 108% decrease, respectively) (Figure 3F). Consistent with our previous observation, no significant change of circulating T cells (Figure 3E) or tumor-resident T cells (Figure 3G) was observed in mice co-engrafted with TA-MSCs or RA treated TA-MSCs compared to mice with control BMSCs.

Overall, our data demonstrated RA treatment significantly blocked IL-17 and IFNγ mediated TA-MSC transformation in promoting tumor growth in melanoma.

### RA Inhibits IL-17-Stimulated Myelocyte-Recruiting Chemokine Expression in BMSCs Through Inhibiting NF-κB Signaling Pathway

**FIGURE 4:**
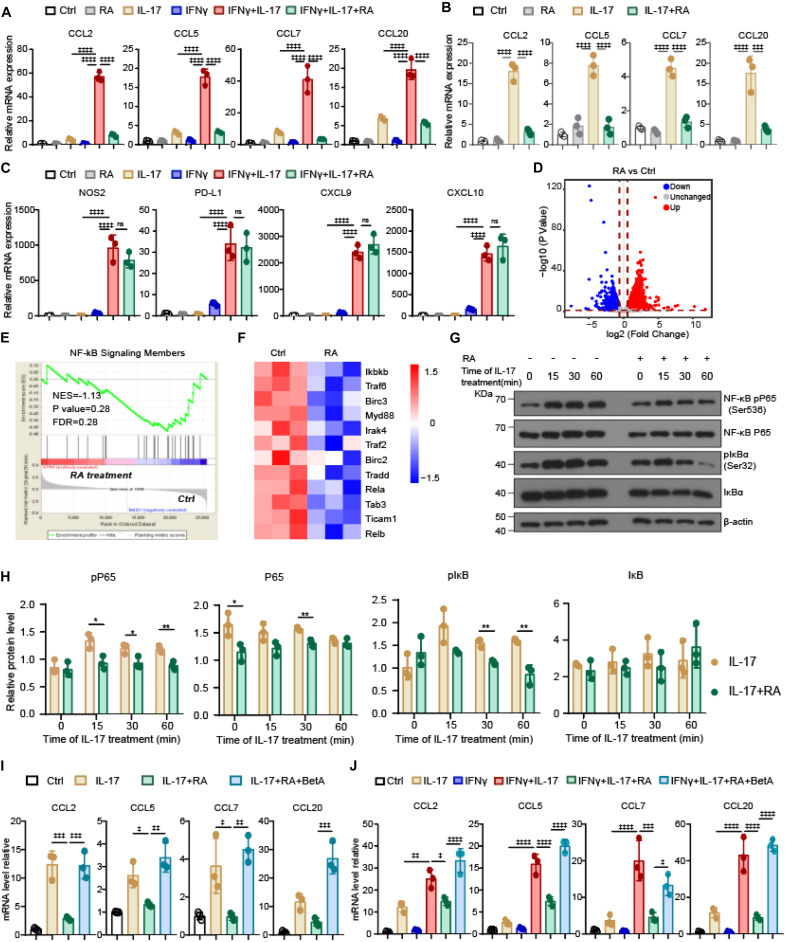
RA inhibits IL-17-stimulated myelocyte-recruiting chemokine expression in BMSCs through inhibiting NF-κB signaling pathway. **(A)** The relative mRNA expression level of myelocyte-recruiting chemokines (CCL2, CCL5, CCL7, and CCL20) in the IL-17 and IFNγ pre-treated BMSCs with or without RA treatment as indicated. **(B)** The relative mRNA expression level of myelocyte-recruiting chemokines in the BMSCs after IL-17 treatment with or without the RA treatment as indicated. **(C)** The relative mRNA expression level of immunoregulatory molecules (NOS2, PD-L1, CXCL9, and CXCL10) in the IL-17 and IFNγ pre-treated BMSCs with or without RA treatment as indicated. **(D)** Volcano plots illustrating the biological and statistical significance of genes between BMSCs with and without RA treatment. Upregulated genes are highlighted in red, unchanged genes are highlighted in gray, and downregulated genes are highlighted in blue. **(E)** Signature enrichment plots from GSEA using NF-κB pathway gene-set in BMSCs treated with and without RA. The enriched *p*-value is derived from Fisher’s exact test. **(F)** A heatmap revealed NF-κB-pathway gene expression changes after RA treatment in BMSCs. The rows show *Z*-scores calculated for each gene. **(G,H)** Immunoblotting analysis of key NF-κB pathway elements in the BMSCs exposed to IL-17 with or without the RA treatment as indicated. **(I)** The relative mRNA expression of myelocyte-recruiting chemokines in BMSCs treated by IL-17, RA, and BetA as indicated. **(J)** The relative mRNA expression of myelocyte-recruiting chemokines in BMSCs treated by IFNγ, IL-17, RA, and BetA as indicated. Data represent mean ± *SD* of 3 independent experiments. * or ^‡^*p* < 0.05, ** or ^‡⁣‡^*p* < 0.01, ^‡⁣‡⁣‡^*p* < 0.001, ^‡⁣‡⁣‡‡^*p* < 0.0001. ns, not significant.

To explore the molecular mechanism that RA suppressed IL-17 and IFNγ mediated TA-MSC transformation, we first analyzed the expression of immunoregulatory molecules in IL-17 and IFNγ transformed TA-MSCs. Intriguingly, RA completely blocked the increase of myelocyte recruiting chemokines expression, including CCL2, CCL5, CCL7, and CCL20, in IL-17 and IFNγ transformed TA-MSCs (Figure 4A). We also noticed that IL-17 treatment alone also slightly increased myelocyte recruiting chemokines expression, which was also completely blocked by RA treatment (87, 89, 90, and 83% reduction) (Figure 4B). However, RA did not inhibit the immunosuppressive molecule expression, including NOS2, PD-L1, CXCL9, CXCL10 (Figure 4C). This indicated that RA inhibited TA-MSC transformation mainly by blocking their ability to recruit myelocytes for tumor-promoting.

To understand the molecular mechanism that RA inhibits IL-17 signaling to suppress myelocyte recruiting chemokine expression, we performed transcription analysis for BMSCs under RA treatment. Our RNA sequencing (RNA-seq) analysis successfully detected 14,423 genes, in which 1,474 genes were upregulated and 1,393 genes were downregulated in BMSCs after RA treatment (Figure 4D). We noticed that NF-κB pathway, which stimulates CCL2 release in TA-MSCs (Katanov et al., 2015), was inhibited in BMSCs upon RA treatment (Figure 4E). Strikingly, major NF-κB pathway elements were downregulated in BMSCs after RA treatment (Figure 4F). Furthermore, RA treatment significantly inhibited IL-17 stimulated phosphorylation of RalA-p65 and IκBα, two key molecules in NF-κB pathway, in BMSCs (Figures 4G,H). This indicated that RA might inhibit NF-κB pathway to suppress the expression of myelocyte recruiting chemokines in IL-17 transformed TA-MSCs. To confirm this, we employed NF-κB specific activator, betulinic acid (BetA) (Kasperczyk et al., 2005), to rescue the suppressed NF-κB signaling in IL-17 transformed TA-MSCs upon RA treatment. Notably, BetA treatment completely rescued the expression of CCL2, CCL5, CCL7, and CCL20 in IL-17 transformed TA-MSCs under RA treatment (Figure 4I). The rescue effect was also observed in IL-17 and IFNγ transformed TA-MSCs (Figure 4J).

Taken together, our data showed that IL-17 activates NF-κB pathway to upregulate myelocyte recruiting chemokines in TA-MSCs, and the TA-MSC transformation was significantly blocked by RA treatment due to inhibition of NF-κB signaling pathway.

### RA Inhibits TNFα Mediated Chemokine Expression in TA-MSCs by Blocking NF-κB Signaling Pathway

**FIGURE 5:**
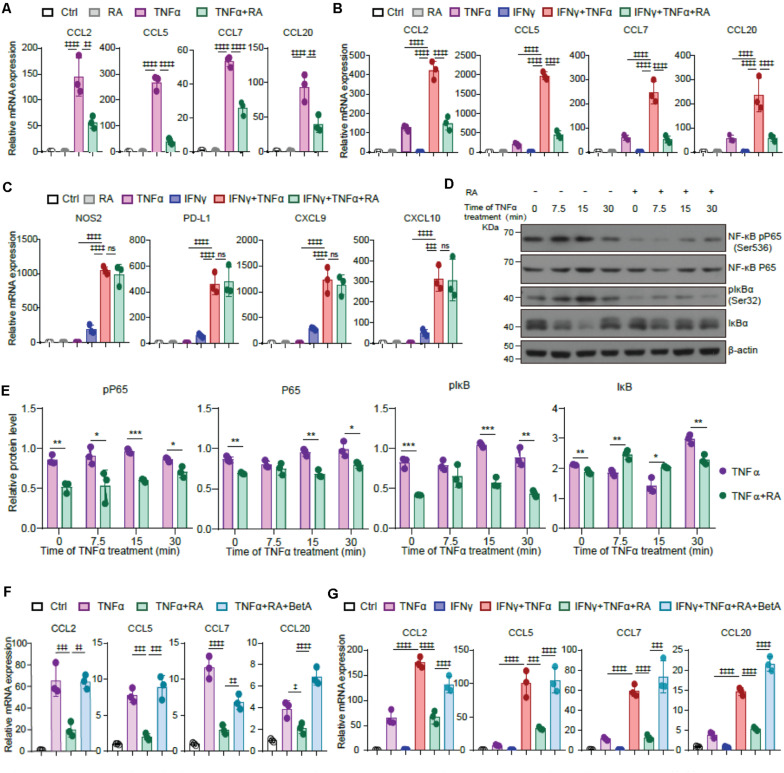
RA inhibits TNFα mediated chemokine expression in TA-MSCs by blocking NF-κB signaling pathway. **(A)** The mRNA expression of myelocyte-recruiting chemokines (CCL2, CCL5, CCL7, and CCL20) in BMSCs treated with TNFα and RA as indicated. **(B,C)** The relative mRNA expression of myelocyte-recruiting chemokines **(B)** and immunoregulatory molecules (NOS2, PD-L1, CXCL9, and CXCL10) **(C)** in the TNFα and IFNγ treated BMSCs with or without the RA treatment as indicated. **(D,E)** Immunoblotting analysis of NF-κB RelA p65, IκBa, and pIκBa (Ser32) in the BMSCs exposed to TNFα and RA as indicated. **(F)** The relative mRNA expression of myelocyte-recruiting chemokines in BMSCs treated by TNFα and RA with or without the BetA as indicated. **(G)** The relative mRNA expression of myelocyte-recruiting chemokines in BMSCs treated by IFNγ, TNFα, RA, and BetA as indicated. Data represent mean ± *SD* of 3 independent experiments. ^∗^*p* < 0.05, ^∗∗^ or ^‡⁣‡^*p* < 0.01, ^∗∗∗^ or ^‡⁣‡⁣‡^*p* < 0.001, ^‡⁣‡⁣‡‡^*p* < 0.0001. ns, not significant.

TNFα transforms TA-MSCs through upregulating myelocyte recruiting chemokines (Ren et al., 2012), therefore, we asked whether RA inhibits TNFα mediated TA-MSC transformation. Interestingly, RA treatment significantly blocked the upregulation of CCL2, CCL5, CCL7, and CCL20 in TNFα treated BMSCs (61, 85, 52, and 58% decrease, respectively) (Figure 5A). Consistently, the RA mediated myelocyte recruiting chemokine expression inhibition was also observed in TNFα and IFNγ treated BMSCs (65, 77, 79, and 76%, decrease, respectively) (Figure 5B). However, we did not observe that RA significantly inhibited immunoregulatory molecules, including NOS2, PD-L1, CXCL9, and CXCL10, which were stimulated by TNFα and IFNγ in BMSCs (Figure 5C). This is consistent with the previous report that TNFα educated TA-MSCs to recruit macrophages to promote tumor growth (Ren et al., 2012).

As NF-κB pathway is critical to CCL family regulation in IL-17 transformed TA-MSCs, we next investigated the role of NF-κB pathway in TNFα mediated TA-MSC transformation. Consistently, we observed that TNFα treatment activated NF-κB pathway in BMSCs, which was evidenced by the activation of NK-κB pP65 and pIκBα, which was coupled with the reduction of IκBα, at 7.5–15 min after TNFα treatment. Notably, RA treatment remarkably attenuated the activation of NFκB pathway in BMSCs under TNFα treatment (Figures 5D,E). More importantly, NF-κB activator, BetA, completely rescued the decrease of CCL2, CCL5, CCL7, and CCL20 under RA treatment in TNFα educated BMSCs (Figure 5F) and BMSCs treated with TNFα and IFNγ simultaneously (Figure 5G).

Taken together, these data demonstrated that RA inhibited NF-κB pathway to suppress the expression of myelocyte chemokines in TNFα educated TA-MSCs.

## Discussion

BMSCs can be transformed into TA-MSCs, which is featured by producing high-level CCR2 ligands to recruit monocytes/macrophages and MDSCs in promoting tumor growth (Ren et al., 2012; Wang Y. et al., 2014; Lin et al., 2016). Tumor proinflammatory cytokine, TNFα, efficiently transforms BMSCs into TA-MSCs and promotes tumor growth in lymphoma, melanoma, and breast carcinoma (Ren et al., 2012; Katanov et al., 2015). IL-17 is involved in inflammatory process and enhances the expression of an immunosuppressive molecule, NOS2, in murine hepatitis (Oukka, 2008; Han et al., 2014). However, unlike innate immune cell generated TNFα, IL-17 is derived from T cells (Oukka, 2008). IL-17 has both pro-tumor and anti-tumor effects. IL-17 inhibits tumor progression and metastasis in melanoma and colon cancer by promoting the function of T cells and NK cells (Kryczek et al., 2009; Martin-Orozco et al., 2009). However, growing evidences show that IL-17 promotes tumor growth in various solid tumors, including melanoma, breast cancer, colon cancer, and hepatocellular carcinoma (Wang et al., 2009; Grivennikov et al., 2012; Coffelt et al., 2015; Gomes et al., 2016). Genetic evidence shows that IL-17 can directly promote proliferation of transformed colonic epithelial cells tumor through its type A receptor (IL-17RA) (Wang K. et al., 2014). Here, we found that IL-17 cooperating with IFNγ to transform TA-MSCs in supporting tumor growth in melanoma. These suggested that blocking IL-17 signaling may inhibit melanoma cells through multiple mechanisms. Out work also suggested that adaptive immune cells can modulate the protumorigenic function of TA-MSCs, which recruits macrophages to support tumor growth (Ren et al., 2012). Intriguingly, we observed that IL-17 treatment alone cannot efficiently transform BMSCs to TA-MSCs, due to the less myelocyte recruiting chemokine expression, and limited ability to recruit macrophages and MDSCs. However, IFNγ remarkably strengthened the myelocyte recruiting chemokines upregulation ability of IL-17, therefore IFNγ and IL-17 synergistically promoted BMSC to TA-MSC transformation. Moreover, TNFα induces cell necroptosis and apoptosis (Locksley et al., 2001; Kalliolias and Ivashkiv, 2016), therefore TNFα transformed TA-MSCs may have limited ability to promote tumor growth. However, IL-17 promotes cell proliferation (Wu et al., 2014), presumably, IL-17 transformed TA-MSCs may have greater efficiency to promote tumor growth. Accordingly, high IL-17 level is observed in colon cancer, skin cancer, and lung cancer patients with poor clinical outcome (Marshall et al., 2016; Razi et al., 2019; Bellone et al., 2020). Recent work showed that IL-17 also regulates the protumorigenic function of cancer-associated fibroblasts (CAFs) (Mucciolo et al., 2021), which are also transformed from normal BMSCs (Quante et al., 2011). Therefore, further studies are warranted to determine the discrepancy between TA-MSCs and CAF in regulating melanoma.

ATRA has revolutionized the treatment of acute promyelocytic leukemia (de Thé, 2018). However, the application of ATRA in solid tumors remains to be explored. RA can inhibit tumor cell proliferation in melanoma (Edward and MacKie, 1989; Zhang and Rosdahl, 2005; Li and Han, 2020) and promote immune surveillance in breast cancer, colorectal cancer, and melanoma by influencing the metabolism of MDSCs, upregulating genes related to immune response, and supporting the survival of tumor-specific CD8^+^ T cells (Guo et al., 2012; Paroni et al., 2020; Sun et al., 2020). Conversely, RA treatment was also reported to benefits tumor progression in sarcoma and chronic lymphocytic leukemia (CLL) by promoting the pro-tumoral differentiation of intertumoral monocytes in sarcoma and increasing CD38 expression in CLL cells (Chen et al., 2018; Devalaraja et al., 2020). Our study showed that RA treatment almost completely inhibited the increase of myelocyte recruiting ability of IL-17 and IFNγ transformed TA-MSCs, although it barely influenced the expression of the immunosuppressive molecules induced by IFNγ. RA treatment successfully inhibited the BMSC to TA-MSC transformation and significantly inhibited tumor growth in melanoma, which opens an avenue for tumor microenvironment targeting therapy.

Both IL-17 and TNFα can activate NF-κB signaling pathway (Sugita et al., 2002; Taniguchi and Karin, 2018) and we confirmed that NF-κB signaling pathway was activated in BMSCs under IL-17 or TNFα treatment. Moreover, NF-κB pathway activation is proved to be important for the paracrine function of tumor-derived-MSCs and cancer-associated fibroblasts in lung cancer and breast cancer in secreting CCL2, IL-6, and IL-8 in the tumor microenvironment (Katanov et al., 2015; Li et al., 2016; Bai et al., 2017; Su et al., 2018). RA is shown to inhibit NF-κB pathway in LPS-stimulated monocytes and renal cells through RARα-STAT1-dependent or TLR4-dependent mechanisms (Austenaa et al., 2009; Sierra-Mondragon et al., 2018). Our finding showed that RA inhibited NF-κB pathway in IL-17- or TNFα-treated BMSCs, which indicates that RA inhibits proinflammatory-factor-mediated BMSC to TA-MSC transformation by inhibiting NF-κB pathway. Indeed, NF-κB pathway activator completely recovered the TA-MSC transformation, which was inhibited by RA treatment in TNFα or IL-17 transformed TA-MSCs. NF-κB promotes tumor growth (Barcellos-de-Souza et al., 2016; Yu et al., 2017). Consistently, our work showed that NF-κB stimulated the tumor supporting function of TA-MSCs. Although IL-17 is considered as a modest activator of NF-κB pathway (Shen and Gaffen, 2008), evidence suggest that IL-17 activates NF-κB through multiple avenues, such as mitogen-activated protein kinase (MAPK) pathway and transforming growth factor β-activated kinase (TAK)1 (Amatya et al., 2017).

Collectively, our study identified IL-17 can educate healthy BMSCs into TA-MSCs, and uncovered a new therapeutic approach to target TA-MSCs by RA. This finding may extend the mechanism and application of RA in tumor therapy.

## Materials and Methods

### Reagents and Mice

Murine IFNγ (315-05-100), IL-17A (210-17) were purchased from PEPROTECH. Murine TNFα (410-MT) was purchased from R&D Systems. Retinoic acid (PHR1187) was purchased from Sigma-Aldrich. Betulinic acid (BetA, HY-10529) was purchased from MedChemExpress. Monoclonal antibodies to CD11b (M1/70), F4/80 (BM8), and Ly6G (17-5931-82) were purchased from eBioscience and Gr-1 (RB6-8C5) was purchased from BioLegend. Primary antibodies for western blotting against P65 (rabbit, 1:1,000, 8,242), pP65 (Ser536) (rabbit, 1:1,000, 3,033), IκBα (rabbit, 1:1,000, 4,812), pIκBα (Ser32) (rabbit, 1:1,000, 2,859), β-actin (rabbit, 1:1,000, 4,970) were purchased from Cell Signaling Technology.

C57BL/6 mice were bred under specific pathogen-free conditions in the animal facility of Sun Yat-sen university. All animal protocols were approved by our Institutional Animal Care and Use Committee.

### Cell Culture

BMSCs were isolated from the tibia and femur bone marrow of C57BL/6 mice following the protocol described in the previous reference (Ren et al., 2012). Cells were maintained in DMEM low-glucose medium (10-014-CVR, CORNING) supplemented with 20% fetal bovine serum (12483020, Gibco), 2% penicillin-streptomycin (SV30010, Invitrogen) and 10 μM ROCK inhibitor (S1049, Selleck) in the adhesive petri dishes. All non-adherent cells were removed after 24 h, and adherent cells were maintained. To obtain MSC clones, cells maintained in 10 cm dishes at 80–90% density were harvested and seeded into 6-well plates at a density of 5 × 10^5^ cells/well. Cells were used before the 3rd passage. B16F0 cells were maintained in DMEM high-glucose medium (10-013-CVR, CORNING) supplemented with 10% FBS and 1% penicillin-streptomycin.

### RNA Isolation and Gene Expression Assay

Before RNA isolation, BMSCs were incubated with or without cytokines of (50 ng ml^–1^ IL-17, 10 ng ml^–1^ IFNγ, and 10 ng ml^–1^ TNFα) or drugs (100 nM RA and 10 μg ml^–1^ BetA), respectively, or jointly for 6 h (Han et al., 2014; Song et al., 2015). Total mRNA was isolated with MagZol^TM^ Reagent (R4801-03, Magen) according to the manufacturer’s instruction. mRNA purity and quantity were determined with NanoDrop (Thermo Scientific) before qPCR and RNA-seq analysis. For Real-Time qPCR, cDNA was synthesized from mRNA by using the TransScript All-in-One First-Strand cDNA Synthesis SuperMix for qPCR (One-Step gDNA Removal) Kit (AT341, Transgen). Quantitative Real-Time PCR was performed on Bio-Rad CFX96 Touch^TM^ Real-Time PCR Detection system with SYBR Green I Master Mix reagent (11203ES03, YEASEN). Sequences of forward and reverse primer pairs are as follows:

**Table:** 

**Gene**	**Forward primer**	**Reverse primer**
	**sequence (5′-3′)**	**sequence (5′-3′)**
Nos2	GTTCTCAGCCCAACAATACAAGA	GTGGACGGGTCGATGTCAC
PD-L1	GCTCCAAAGGACTTGTACGTG	TGATCTGAAGGGCAGCATTTC
Cxcl9	TCCTTTTGGGCATCATCTTCC	TTTGTAGTGGATCGTGCCTCG
Cxcl10	CCAAGTGCTGCCGTCATTTTC	GGCTCGCAGGGATGATTTCAA
CCL2	TTAAAAACCTGGATCGGAACCAA	GCATTAGCTTCAGATTTACGGGT
CCL5	GCTGCTTTGCCTACCTCTCC	TCGAGTGACAAACACGACTGC
CCL7	GCTGCTTTCAGCATCCAAGTG	CCAGGGACACCGACTACTG
CCL20	GCCTCTCGTACATACAGACGC	CCAGTTCTGCTTTGGATCAGC
β-actin	GGCTGTATTCCCCTCCATCG	CCAGTTGGTAACAATGCCATGT

### Tumor Transplantation

1 × 10^5^ BMSCs which were pre-treated with or without 50 ng ml^–1^ IL-17, 10 ng ml^–1^ IFNγ, and 100 nM RA, respectively, or jointly for 12 h before subcutaneously injection with 2.5 × 10^5^ B16F0 into recipient C57BL/6 mice. Tumor size and weight were measured at various time points. Peripheral blood was collected on the 6th and 12th day and resultant tumors were harvested on the 12th day after tumor cell inoculation for further analysis.

### Flow Cytometry

For cell population analysis, cells isolated from peripheral blood and tumors were suspended in staining buffer (PBS, 2% FBS) at a concentration of 2 × 10^6^ cells ml^–1^ and 100 ml of suspension was incubated with fluorescently labeled antibodies for 1 h on ice. Macrophages were gated as CD11b^+^ F4/80^+^. Neutrophils were gated as CD11b^+^ Ly6G^+^. Monocytes were gated as CD11b^+^Gr-1^+^ Ly6G^–^. MDSCs were gated as CD11b^+^Gr-1^+^. Analyses were performed using a flow cytometer (Attune NxT; Thermo Fisher). The immune cell frequency was calculated as the frequency of each immune cell population in total nucleated cells in peripheral blood or total resident nucleated blood cells from tumor site.

### Western Blotting

For immunoblotting analysis, BMSCs incubated with 50 ng ml^–1^ IL-17 for 15, 30, and 60 min or 10 ng ml^–1^ TNFα for 7.5, 15, 30 min were pre-challenged by 100 nM RA for 6 h. Cells were washed with ice-cold PBS, harvested and lysed for 15 min by lysis buffer containing 0.5% TritonX-100 (T9284, Sigma), 20 mM Hepes pH7.4 (H-4034, Sigma), 150 mM NaCl (A100241, Sangon Biotech), 12.5 mM β-glycerophosphate (A500486, Sangon Biotech), 1.5 mM MgCl_2_ (M4880, Sigma), 2 mM EGTA (A600077, Sangon Biotech), and a cocktail of protease inhibitors, Na_3_VO_4_ (A600869, Sangon Biotech), NaF (A500850, Sangon Biotech), and PMSF (A610425, Sangon Biotech). Equal amounts of protein extracts were resolved in 10% SDS-PAGE and transferred to PVDF membranes (IPVH00010, Merck Millipore). The membranes were blocked with 5% non-fat milk in Tris-buffered saline with Tween-20 (TBST, pH 7.6) for 1 h at room temperature before incubated overnight with the primary antibodies (p65 1:1,000, pp65 (Ser536) 1:1,000, IκBα 1:1,000, pIκBα (Ser32) 1:1,000, β-actin 1:1,000) at 4°C and then incubated with the secondary antibodies (rabbit, 1:10,000, W401B, Promega) for 1 h at room temperature. Finally, the blots were detected by enhanced chemiluminescent reagents (Millipore).

### Gene Set Enrichment Analysis (GSEA)

RNA of control MSC and RA pretreated MSCs (100 nM RA for 24 h) were used for RNAseq analysis. Raw data.QZ files were imported into GSEA 3.0 software where background correction and normalization were performed with standard default settings. The.QZ files were combined into one.gct file in GenePattern, then imported into GSEA along with a matching phenotype label file (.cls). GSEA analysis was run with the following parameters: number of permutations = 1,000, collapse dataset to gene symbols = false, permutation type = gene_set, plot graphs for the top sets of each phenotype = 150 (default = 20), gene sets database = h.all.v6.0 symbols.gmt (all hallmarks, version 6), with a phenotype comparison of RA pre-treatment vs. control BMSCs. Leading edge analysis was completed on the Hallmark GSEA output with NF-κB signaling hallmark gene set.

### Statistical Analysis

The statistical analysis was performed using GraphPad Prism 8.0 software. Two-tailed Student’s *t* tests were used for the comparison between two groups (*p < 0.05, **p < 0.01, and ***p < 0.001) and the one-way ANOVAs with Tukey’s multiple comparison tests were used for the comparison between more than two groups (^‡^*p* < 0.05, ^‡‡^*p* < 0.01, ^‡⁣‡‡^*p* < 0.001, and ^‡⁣‡⁣‡‡^*p* < 0.0001). The two-way ANOVAs with Tukey’s multiple comparison tests were used for comparison between more than two groups at various time points (^‡^*p* < 0.05, ^‡‡^*p* < 0.01, ^‡⁣‡‡^*p* < 0.001, and ^‡⁣‡⁣‡‡^*p* < 0.0001). All data are expressed as mean ± *SD*.

## Data Availability Statement

The accession number for the RNA-seq data reported in our manuscript is GEO: GSE169145.

## Ethics Statement

The animal study was reviewed and approved by the Institutional Animal Care and Use Committee, SYSU.

## Author Contributions

QL, MiZ, and QX designed and performed most of the experiments and analyzed the data. SX, YL, JC, LY, and LW contributed to animal experiments and all the transcriptional assay. LM, DL, and LJ contributed to the discussion. QL, MiZ, and MeZ wrote the manuscript. MeZ supervised the project. All authors contributed to the article and approved the submitted version.

## Conflict of Interest

The authors declare that the research was conducted in the absence of any commercial or financial relationships that could be construed as a potential conflict of interest.

## References

[B1] Abu J. Batuwangala M. Herbert K. Symonds P. (2005). Retinoic acid and retinoid receptors: potential chemopreventive and therapeutic role in cervical cancer. *Lancet Oncol.* 6 712–720. 10.1016/s1470-2045(05)70319-3 16129372

[B2] Amatya N. Garg A. Gaffen S. (2017). IL-17 signaling: the Yin and the Yang. *Trends Immunol.* 38 310–322. 10.1016/j.it.2017.01.006 28254169 PMC5411326

[B3] Austenaa L. Carlsen H. Hollung K. Blomhoff H. Blomhoff R. (2009). Retinoic acid dampens LPS-induced NF-kappaB activity: results from human monoblasts and in vivo imaging of NF-kappaB reporter mice. *J. Nutr. Biochem.* 20 726–734. 10.1016/j.jnutbio.2008.07.002 18926686

[B4] Bai X. Xi J. Bi Y. Zhao X. Bing W. Meng X. (2017). TNF-α promotes survival and migration of MSCs under oxidative stress via NF-κB pathway to attenuate intimal hyperplasia in vein grafts. *J. Cell. Mol. Med.* 21 2077–2091. 10.1111/jcmm.13131 28266177 PMC5571532

[B5] Barcellos-de-Souza P. Comito G. Pons-Segura C. Taddei M. Gori V. Becherucci V. (2016). Mesenchymal stem cells are recruited and activated into carcinoma-associated fibroblasts by prostate cancer microenvironment-derived TGF-β1. *Stem Cells* 34 2536–2547. 10.1002/stem.2412 27300750

[B6] Beckermann B. Kallifatidis G. Groth A. Frommhold D. Apel A. Mattern J. (2008). VEGF expression by mesenchymal stem cells contributes to angiogenesis in pancreatic carcinoma. *Br. J. Cancer* 99 622–631. 10.1038/sj.bjc.6604508 18665180 PMC2527820

[B7] Bellone M. Brevi A. Huber S. (2020). Microbiota-propelled T helper 17 cells in inflammatory diseases and cancer. *Microbiol. Mol. Biol. Rev.* 84:e00064-19. 10.1128/MMBR.00064-19 32132244 PMC7062199

[B8] Bolis M. Paroni G. Fratelli M. Vallerga A. Guarrera L. Zanetti A. (2020). All-trans retinoic acid stimulates viral mimicry, interferon responses and antigen presentation in breast-cancer cells. *Cancers* 12:1169. 10.3390/cancers12051169 32384653 PMC7281473

[B9] Chen L. Diao L. Yang Y. Yi X. Rodriguez B. Li Y. (2018). CD38-mediated immunosuppression as a mechanism of tumor cell escape from PD-1/PD-L1 blockade. *Cancer Discov.* 8 1156–1175. 10.1158/2159-8290.cd-17-1033 30012853 PMC6205194

[B10] Coffelt S. Kersten K. Doornebal C. Weiden J. Vrijland K. Hau C. (2015). IL-17-producing γδ T cells and neutrophils conspire to promote breast cancer metastasis. *Nature* 522 345–348. 10.1038/nature14282 25822788 PMC4475637

[B11] Cunningham T. Duester G. (2015). Mechanisms of retinoic acid signalling and its roles in organ and limb development. *Nat. Rev. Mol. Cell Biol.* 16 110–123. 10.1038/nrm3932 25560970 PMC4636111

[B12] de Thé H. (2018). Differentiation therapy revisited. *Nat. Rev. Cancer* 18 117–127. 10.1038/nrc.2017.103 29192213

[B13] Devalaraja S. To T. Folkert I. Natesan R. Alam M. Li M. (2020). Tumor-derived retinoic acid regulates intratumoral monocyte differentiation to promote immune suppression. *Cell* 180 1098–1114.e1016. 10.1016/j.cell.2020.02.042 32169218 PMC7194250

[B14] Edward M. MacKie R. (1989). Retinoic acid-induced inhibition of lung colonization and changes in the synthesis and properties of glycosaminoglycans of metastatic B16 melanoma cells. *J. Cell Sci.* 94(Pt 3) 537–543. 2517293 10.1242/jcs.94.3.537

[B15] Elias K. Laurence A. Davidson T. Stephens G. Kanno Y. Shevach E. (2008). Retinoic acid inhibits Th17 polarization and enhances FoxP3 expression through a Stat-3/Stat-5 independent signaling pathway. *Blood* 111 1013–1020. 10.1182/blood-2007-06-096438 17951529 PMC2214761

[B16] Friedenstein A. Chailakhyan R. Gerasimov U. (1987). Bone marrow osteogenic stem cells: in vitro cultivation and transplantation in diffusion chambers. *Cell Tissue Kinet.* 20 263–272. 10.1111/j.1365-2184.1987.tb01309.x 3690622

[B17] Friedenstein A. Gorskaja J. Kulagina N. (1976). Fibroblast precursors in normal and irradiated mouse hematopoietic organs. *Exp. Hematol.* 4 267–274. 976387

[B18] Friedenstein A. Piatetzky-Shapiro I. Petrakova K. (1966). Osteogenesis in transplants of bone marrow cells. *J. Embryol. Exp. Morphol.* 16 381–390. 5336210

[B19] Gomes A. Teijeiro A. Burén S. Tummala K. Yilmaz M. Waisman A. (2016). Metabolic inflammation-associated IL-17A causes non-alcoholic steatohepatitis and hepatocellular carcinoma. *Cancer Cell* 30 161–175. 10.1016/j.ccell.2016.05.020 27411590

[B20] Grivennikov S. Wang K. Mucida D. Stewart C. Schnabl B. Jauch D. (2012). Adenoma-linked barrier defects and microbial products drive IL-23/IL-17-mediated tumour growth. *Nature* 491 254–258. 10.1038/nature11465 23034650 PMC3601659

[B21] Guo Y. Pino-Lagos K. Ahonen C. Bennett K. Wang J. Napoli J. (2012). A retinoic acid–rich tumor microenvironment provides clonal survival cues for tumor-specific CD8(+) T cells. *Cancer Res.* 72 5230–5239. 10.1158/0008-5472.can-12-1727 22902413 PMC3766319

[B22] Han X. Yang Q. Lin L. Xu C. Zheng C. Chen X. (2014). Interleukin-17 enhances immunosuppression by mesenchymal stem cells. *Cell Death Differ.* 21 1758–1768. 10.1038/cdd.2014.85 25034782 PMC4211372

[B23] He D. Li H. Yusuf N. Elmets C. Li J. Mountz J. (2010). IL-17 promotes tumor development through the induction of tumor promoting microenvironments at tumor sites and myeloid-derived suppressor cells. *J. Immunol.* 184 2281–2288. 10.4049/jimmunol.0902574 20118280 PMC3179912

[B24] Jiang W. Xu J. (2020). Immune modulation by mesenchymal stem cells. *Cell Prolif.* 53:e12712. 10.1111/cpr.12712 31730279 PMC6985662

[B25] Kalliolias G. Ivashkiv L. (2016). TNF biology, pathogenic mechanisms and emerging therapeutic strategies. *Nat. Rev. Rheumatol.* 12 49–62. 10.1038/nrrheum.2015.169 26656660 PMC4809675

[B26] Kasperczyk H. La Ferla-Brühl K. Westhoff M. Behrend L. Zwacka R. Debatin K. (2005). Betulinic acid as new activator of NF-kappaB: molecular mechanisms and implications for cancer therapy. *Oncogene* 24 6945–6956. 10.1038/sj.onc.1208842 16007147

[B27] Katanov C. Lerrer S. Liubomirski Y. Leider-Trejo L. Meshel T. Bar J. (2015). Regulation of the inflammatory profile of stromal cells in human breast cancer: prominent roles for TNF-α and the NF-κB pathway. *Stem Cell Res. Ther.* 6:87. 10.1186/s13287-015-0080-7 25928089 PMC4469428

[B28] Kfoury Y. Scadden D. (2015). Mesenchymal cell contributions to the stem cell niche. *Cell Stem Cell* 16 239–253. 10.1016/j.stem.2015.02.019 25748931

[B29] Kryczek I. Wei S. Szeliga W. Vatan L. Zou W. (2009). Endogenous IL-17 contributes to reduced tumor growth and metastasis. *Blood* 114 357–359. 10.1182/blood-2008-09-177360 19289853 PMC2714210

[B30] Kumar V. Patel S. Tcyganov E. Gabrilovich D. (2016). The nature of myeloid-derived suppressor cells in the tumor microenvironment. *Trends Immunol.* 37 208–220. 10.1016/j.it.2016.01.004 26858199 PMC4775398

[B31] Li C. Han X. (2020). Co-delivery of dacarbazine and all-trans retinoic acid (ATRA) using lipid nanoformulations for synergistic antitumor efficacy against malignant melanoma. *Nanoscale Res. Lett.* 15:113. 10.1186/s11671-020-3293-3 32430641 PMC7237551

[B32] Li X. Wang S. Zhu R. Li H. Han Q. Zhao R. (2016). Lung tumor exosomes induce a pro-inflammatory phenotype in mesenchymal stem cells via NFκB-TLR signaling pathway. *J. Hematol. Oncol.* 9:42. 10.1186/s13045-016-0269-y 27090786 PMC4836087

[B33] Lin L. Du L. Cao K. Huang Y. Yu P. Zhang L. (2016). Tumour cell-derived exosomes endow mesenchymal stromal cells with tumour-promotion capabilities. *Oncogene* 35 6038–6042. 10.1038/onc.2016.131 27132512 PMC5116561

[B34] Locksley R. Killeen N. Lenardo M. (2001). The TNF and TNF receptor superfamilies: integrating mammalian biology. *Cell* 104 487–501. 10.1016/s0092-8674(01)00237-9 11239407

[B35] Marshall E. A. Ng K. W. Kung S. H. Conway E. M. Martinez V. D. Halvorsen E. C. (2016). Emerging roles of T helper 17 and regulatory T cells in lung cancer progression and metastasis. *Mol. Cancer* 15:27. 10.1186/s12943-016-0551-1 27784305 PMC5082389

[B36] Martin-Orozco N. Muranski P. Chung Y. Yang X. Yamazaki T. Lu S. (2009). T helper 17 cells promote cytotoxic T cell activation in tumor immunity. *Immunity* 31 787–798. 10.1016/j.immuni.2009.09.014 19879162 PMC2787786

[B37] McLean K. Gong Y. Choi Y. Deng N. Yang K. Bai S. (2011). Human ovarian carcinoma–associated mesenchymal stem cells regulate cancer stem cells and tumorigenesis via altered BMP production. *J. Clin. Invest.* 121 3206–3219. 10.1172/jci45273 21737876 PMC3148732

[B38] Miossec P. Korn T. Kuchroo V. (2009). Interleukin-17 and type 17 helper T cells. *N. Engl. J. Med.* 361 888–898. 10.1056/NEJMra0707449 19710487

[B39] Mucciolo G. Curcio C. Roux C. Li W. Capello M. Curto R. (2021). IL17A critically shapes the transcriptional program of fibroblasts in pancreatic cancer and switches on their protumorigenic functions. *Proc. Natl. Acad. Sci. U.S.A.* 118:e2020395118. 10.1073/pnas.2020395118 33526692 PMC8017922

[B40] Mucida D. Park Y. Kim G. Turovskaya O. Scott I. Kronenberg M. (2007). Reciprocal TH17 and regulatory T cell differentiation mediated by retinoic acid. *Science* 317 256–260. 10.1126/science.1145697 17569825

[B41] Oukka M. (2008). Th17 cells in immunity and autoimmunity. *Ann. Rheum. Dis.* 67(Suppl. 3) iii26–iii29. 10.1136/ard.2008.098004 19022809

[B42] Paroni G. Zanetti A. Barzago M. Kurosaki M. Guarrera L. Fratelli M. (2020). Retinoic acid sensitivity of triple-negative breast cancer cells characterized by constitutive activation of the notch1 pathway: the role of Rarβ. *Cancers* 12:3027. 10.3390/cancers12103027 33081033 PMC7650753

[B43] Pietras K. Ostman A. (2010). Hallmarks of cancer: interactions with the tumor stroma. *Exp. Cell Res.* 316 1324–1331. 10.1016/j.yexcr.2010.02.045 20211171

[B44] Qian B. Pollard J. (2010). Macrophage diversity enhances tumor progression and metastasis. *Cell* 141 39–51. 10.1016/j.cell.2010.03.014 20371344 PMC4994190

[B45] Quante M. Tu S. Tomita H. Gonda T. Wang S. Takashi S. (2011). Bone marrow-derived myofibroblasts contribute to the mesenchymal stem cell niche and promote tumor growth. *Cancer Cell* 19 257–272. 10.1016/j.ccr.2011.01.020 21316604 PMC3060401

[B46] Razi S. Baradaran Noveiry B. Keshavarz-Fathi M. Rezaei N. (2019). IL-17 and colorectal cancer: from carcinogenesis to treatment. *Cytokine* 116 7–12. 10.1016/j.cyto.2018.12.021 30684916

[B47] Ren G. Zhang L. Zhao X. Xu G. Zhang Y. Roberts A. (2008). Mesenchymal stem cell-mediated immunosuppression occurs via concerted action of chemokines and nitric oxide. *Cell Stem Cell* 2 141–150. 10.1016/j.stem.2007.11.014 18371435

[B48] Ren G. Zhao X. Wang Y. Zhang X. Chen X. Xu C. (2012). CCR2-dependent recruitment of macrophages by tumor-educated mesenchymal stromal cells promotes tumor development and is mimicked by TNFα. *Cell Stem Cell* 11 812–824. 10.1016/j.stem.2012.08.013 23168163 PMC3518598

[B49] Shen F. Gaffen S. (2008). Structure-function relationships in the IL-17 receptor: implications for signal transduction and therapy. *Cytokine* 41 92–104. 10.1016/j.cyto.2007.11.013 18178098 PMC2667118

[B50] Sierra-Mondragon E. Molina-Jijon E. Namorado-Tonix C. Rodríguez-Muñoz R. Pedraza-Chaverri J. Reyes J. (2018). All-trans retinoic acid ameliorates inflammatory response mediated by TLR4/NF-κB during initiation of diabetic nephropathy. *J. Nutr. Biochem.* 60 47–60. 10.1016/j.jnutbio.2018.06.002 30193155

[B51] Song X. Dai D. He X. Zhu S. Yao Y. Gao H. (2015). Growth factor FGF2 cooperates with interleukin-17 to repair intestinal epithelial damage. *Immunity* 43 488–501. 10.1016/j.immuni.2015.06.024 26320657

[B52] Su S. Chen J. Yao H. Liu J. Yu S. Lao L. (2018). CD10GPR77 cancer-associated fibroblasts promote cancer formation and chemoresistance by sustaining cancer stemness. *Cell* 172 841–856.e816. 10.1016/j.cell.2018.01.009 29395328

[B53] Sugita S. Kohno T. Yamamoto K. Imaizumi Y. Nakajima H. Ishimaru T. (2002). Induction of macrophage-inflammatory protein-3alpha gene expression by TNF-dependent NF-kappaB activation. *J. Immunol.* 168 5621–5628. 10.4049/jimmunol.168.11.5621 12023359

[B54] Sun H. Chen J. Wu W. Yang Y. Xu Y. Yu X. (2020). Retinoic acid synthesis deficiency fosters the generation of polymorphonuclear myeloid-derived suppressor cells in colorectal cancer. *Cancer Immunol. Res.* 9 20–33. 10.1158/2326-6066.cir-20-0389 33177108

[B55] Taniguchi K. Karin M. (2018). NF-κB, inflammation, immunity and cancer: coming of age. *Nat. Rev. Immunol.* 18 309–324. 10.1038/nri.2017.142 29379212

[B56] Uccelli A. Moretta L. Pistoia V. (2008). Mesenchymal stem cells in health and disease. *Nat. Rev. Immunol.* 8 726–736. 10.1038/nri2395 19172693

[B57] Wang K. Kim M. Di Caro G. Wong J. Shalapour S. Wan J. (2014). Interleukin-17 receptor a signaling in transformed enterocytes promotes early colorectal tumorigenesis. *Immunity* 41 1052–1063. 10.1016/j.immuni.2014.11.009 25526314 PMC4272447

[B58] Wang L. Yi T. Kortylewski M. Pardoll D. Zeng D. Yu H. (2009). IL-17 can promote tumor growth through an IL-6-Stat3 signaling pathway. *J. Exp. Med.* 206 1457–1464. 10.1084/jem.20090207 19564351 PMC2715087

[B59] Wang Y. Chen X. Cao W. Shi Y. (2014). Plasticity of mesenchymal stem cells in immunomodulation: pathological and therapeutic implications. *Nat. Immunol.* 15 1009–1016. 10.1038/ni.3002 25329189

[B60] Wu P. Wu D. Ni C. Ye J. Chen W. Hu G. (2014). γδT17 cells promote the accumulation and expansion of myeloid-derived suppressor cells in human colorectal cancer. *Immunity* 40 785–800. 10.1016/j.immuni.2014.03.013 24816404 PMC4716654

[B61] Yu P. Huang Y. Xu C. Lin L. Han Y. Sun W. (2017). Downregulation of CXCL12 in mesenchymal stromal cells by TGFβ promotes breast cancer metastasis. *Oncogene* 36 840–849. 10.1038/onc.2016.252 27669436 PMC5311419

[B62] Zhang H. Rosdahl I. (2005). Expression profiles of Id1 and p16 proteins in all-trans-retinoic acid-induced apoptosis and cell cycle re-distribution in melanoma. *Cancer Lett.* 217 33–41. 10.1016/j.canlet.2004.07.033 15596294

